# The impact of appearance-focussed social media and self-objectification on mood

**DOI:** 10.1371/journal.pone.0355465

**Published:** 2026-09-09

**Authors:** Samantha M. Jones, Faith A. Pasternak, Erin A. Heerey

**Affiliations:** Department of Psychology, Western University, London, Ontario, Canada; University of Cagliari: Universita degli Studi Di Cagliari, ITALY

## Abstract

Viewing appearance-focussed social media (e.g., thinspiration, fitspiration, body positivity) is associated with increased self-objectification in women. However, it is unclear how increases in self-objectification directly relate to momentary differences in mood when viewing different types of social media content, and whether a general tendency to self-objectify enhances vulnerability to negative moods in this context. The current study used a within-subjects experimental design to examine momentary differences in self-objectification and mood across five categories of TikTok videos (three appearance-focussed; two neutral). 323 women and feminine-identifying people reported on their general disposition to self-objectify before viewing a randomized set of 20 TikTok videos. After each video, participants reported state levels of self-objectification along with positive and negative affect. Individuals with a greater tendency to self-objectify reported more state self-objectification after viewing thinspiration and body positive content and were more vulnerable to increases in negative mood after viewing thinspiration. Interestingly, increases in state self-objectification were linked to increases in positive mood after viewing body positive content. Trait self-objectification had no effect on positive mood across video categories. These results shed light on the conflicting effects of self-objectification on social media induced mood and self-objectification.

## Introduction

Self-objectification occurs when people feel their value is determined by their appearance, that is, they view themselves and their bodies as objects [1]. The concept of self-objectification stems from Objectification Theory which posits that women’s views of themselves are deeply intertwined with their perceptions of how others view them [2]. When self-objectification experiences become more prevalent and consistent across situations, this is known as “trait” self-objectification [1]. Trait self-objectification is associated with negative consequences, including body shame, depressive symptoms, and disordered eating behaviours [3–6]. Interestingly, people with a greater disposition to self-objectify report spending more time on social media [7]. Consequently, increased daily social media use is associated with greater self-objectifying thoughts [8]. The established link between social media and self-objectification may in part be due to the pervasiveness of objectifying content across social media platforms.

Although objectifying content takes many forms on social media, it often falls within appearance-focussed content categories such as *thinspiration*, *fitspiration,* and *body positivity*. *Thinspiration* depicts a thin-ideal and often contains objectifying images of thin bodies and messages that encourage weight loss [9]. Similarly, *fitspiration*, contains both objectifying images and fat-loss promotion messages, however, it also promotes muscle gain via images or videos depicting bodies that are both thin *and* muscular [10]. Exposure to both *thinspiration* and *fitspiration*, so called “appearance-ideal” content, have been found to be associated with reductions in mood, increases in body dissatisfaction, and “state” or momentary self-objectification [11–13]. However, the findings surrounding fitspiration have been somewhat inconsistent, with some studies finding no effect of viewing fitspiration content on mood or self-objectification [14,15].

A contrasting phenomenon, *body positivity,* depicts images of a variety of body types and promotes body acceptance by challenging thin/fit ideals [16]. Unlike *thinspiration* and *fitspiration*, body positive content has been associated with benefits such as increased mood and body satisfaction [11,17]. However, researchers have begun to question the seemingly beneficial nature of body positivity, as images and videos in this content category also depict objectifying images of women [18,19], and exposure to this content has been linked with reports of enhanced state self-objectification [11]. The varying effects that different appearance-focussed social media contents have on mood suggest that state self-objectification may be linked with either positive or negative mood outcomes depending on the content type. The effect of state self-objectification on mood when viewing appearance-focussed social media may be particularly strong for those with a greater tendency to self-objectify.

Accordingly, women high in trait self-objectification appear more likely to self-objectify after viewing appearance-focussed content [20] possibly because the tendency to think in this way often involves comparing oneself to a specific standard of beauty [21]. The *circle of objectification* theory posits that self-objection drives women to compare their bodies to others in an objectifying manner which in turn results in greater self-objectification [22]. Thus, those higher in trait self-objectification may be more likely to experience increases in state self-objectification when viewing appearance-focussed social media content and also more likely to seek out such content. However, the extent to which one’s disposition to self-objectify impacts their mood when viewing appearance-focussed social media content has yet to be examined.

The current study tests associations between trait/state self-objectification and mood after viewing a variety of both appearance-focussed and non-appearance-focussed social media videos. Previous research has found that appearance-focussed social media content is associated with increases in self-objectification, which is robustly associated with negative outcomes such as decreases in mood [e.g., [4]]. Despite overwhelming evidence that self-objectification may be a harmful thought pattern, research in the body positivity movement suggests that despite increasing self-objectification, people feel positively after engaging with such content. Thus, we will examine whether increased state levels of self-objectification after viewing body positive content are directly related to the increases in mood previously observed in the literature. Specifically, this study aims to provide a stronger understanding of how and under what conditions appearance-focussed social media has harmful versus beneficial effects on one’s self-concept (i.e., self-objectification) and how this relates to mood outcomes. Additionally, we seek to build upon previous research and determine whether individuals with a predisposition to self-objectify are more vulnerable to the effects of appearance-focussed content. We tested the following hypotheses:

H1. Viewing appearance-focussed social media will be associated with greater state self-objectification compared to viewing non-appearance focussed social media. We expect video category and trait self-objectification scores to interact, such that the effect of content category on state self-objectification will be stronger for those higher in trait self-objectification.

H2. Viewing thinspiration and fitspiration content will be associated with greater negative mood. We expect that both trait-level self-objectification and post-video levels of state self-objectification to interact with content category. Specifically, we expect that the effect of content category (thinspiration and fitspiration) on negative mood will be stronger for those higher in trait self-objectification (H2A) and those that report greater post-video state levels of self-objectification (H2B).

H3. Viewing body positive content will be associated with greater positive mood. We expect both trait-level self-objectification and post-video levels of state self-objectification to interact with content category. Specifically, we expect that the effect of content category (body positivity) will be strengthened by both trait-level self-objectification (H3A) and post-video state levels of self-objectification (H3B).

## Methods

The current study was approved by the university’s non-medical Research Ethics Board prior to data collection (REB#: 121891). Recruitment began on 24/11/2022 and concluded on 03/08/2023. Participants were recruited via a mass email sent to all students (undergraduate, graduate, and professional) at a large university in Southwestern Ontario, Canada. The email included a link for participants to complete the survey online. In total, 685 participants consented to the study. Participants were given the opportunity to enter a gift-card draw in exchange for participating. As the appearance-focussed videos depicted bodies of women or feminine-presenting people, we sought to include only individuals that might directly relate to the feminine body ideals or experience standards of beauty promoted (e.g., thin-ideal) and challenged (e.g., body positivity) within the content presented during the study [e.g., [23]]. Accordingly, we only included women and feminine-identifying (e.g., trans-femme) people and removed 41 men and/or masculine-identifying people. As pilot-testing indicated the survey would take a minimum of 10 minutes to complete (if all videos were watched fully), we removed 163 participants with completion times of under 10 minutes. Finally, we removed 110 participants who completed fewer than 20% of the video ratings or survey questionnaires and 48 participants who had more than 75% missing data on post-video ratings on the positive and/or negative affect scale. Thus, the final sample (*N* = 323) included 318 cisgender women, 2 transgender women, 1 feminine-presenting transgender individual and 2 people who preferred not to disclose whether they were trans- or cisgender but did identify as feminine-presenting. Sixty-four percent of participants identified as heterosexual and 64% identified as white (Table 1).

**Table 1:** Demographic characteristics of full sample.

		*N*	*Proportion*
Age			
	17-19	96	29.72%
	20-25	168	52.01%
	26-30	39	12.07%
	30+	2	<1%
Sexual Orientation			
	Bisexual	66	20.43%
	Pansexual	8	2.48%
	Gay/Lesbian	9	2.79%
	Asexual	5	1.55%
	Heterosexual	206	63.78%
	More than one orientation listed	12	3.72%
	Other orientation not listed above	8	2.48%
	Prefer not to say/chose not to answer	9	2.79%
Body Size			
	Below average	48	14.86%
	Average	167	51.70%
	Above average	76	23.53%
	Chose not to answer	32	9.91%
Race/Ethnicity			
	Black	7	2.17%
	East Asian	37	11.46%
	Indigenous	1	<1%
	Latina/Latino	3	<1%
	Middle Eastern	4	1.24%
	Pacific Islander or Native Hawaiian	3	<1%
	South Asian	29	8.98%
	White	208	64.40%
	More than one race/ethnicity listed	26	8.05%
	Other race/ethnicity not specified	4	1.24%
	Chose not to answer	1	<1%

The survey began with a demographic questionnaire (e.g., gender identity, age, etc.) followed by measures of trait self-objectification, masculinity/femininity, and TikTok use. Participants then watched 20 randomly selected and ordered TikTok videos that included four videos from each of five categories (thinspiration, fitspiration, body positivity, general positive, and neutral). After each video, participants completed brief mood (i.e., positive and negative affect) and state self-objectification measures.

### Materials

Trait self-objectification was measured using the 14-item Self-Objectification Beliefs and Behaviours Scale [SOBBS; [24]] which showed good internal reliability within the sample (α = 0.88). Participants responded to each item (e.g., “My physical appearance is more important than my physical abilities”) using a 5-point Likert Scale ranging from (1) strongly disagree to (5) strongly agree. Person-mean imputation was used for participants (*n* = 11) who had less than 20% missing data on the SOBBS.

Three researchers familiar with definitions of thinspiration, fitspiration and body positive content selected over 50 TikTok videos in each category to be randomly presented to participants (see S1 Text for details, including a discussion of diversity of the visible content creators). Only videos that were between 10–30 seconds long and depicted bodies of women and feminine-presenting people were included. We excluded videos with a primary focus on eating behaviours as this study examines appearance-focussed content. In addition to collecting appearance-focussed content, researchers collected 1) videos that did not contain images of people’s bodies that were thought to either boost positive mood (i.e., cute/funny animal videos), as these are frequent elements in people’s social media feeds; and 2) videos with a focus on skills that were designed neither to increase nor to decrease mood (i.e., neutral videos, depicting content such as cement pouring, cleaning, painting, etc.). The resulting categories of videos were (1) thinspiration, (2) fitspiration, (3) body positive, (4) non-appearance-focussed positive (“positive”), and (5) non-appearance-focussed neutral (“neutral”). To ensure that participants interpreted the videos as expected, they classified each stimulus according to the best fitting category/categories at the end of the task (S1 Text).

After each video, participants reported on their mood and state self-objectification. Using two slider bars participants indicated how each video made them feel in terms of (1) positive/good feeling and (2) negative/bad feeling. Each slider bar ranged from 0 (“Not at all”) to 100 (“Extremely”). Positive and negative valence were significantly and negatively correlated (*ρ =* −0.23, *p* < 0.001). Self-objectification is often measured by the extent to which participants think of their appearance over their skills and abilities [1]. Thus, to measure state self-objectification, participants answered the following three questions using a 1–5 Likert scale ranging from (1) “Not at all” to (5) “Extremely”: (1) To what extent does this video make you think about the things you do like about your body? (2) To what extent does this video make you think about the things you do **not** like about your body? (3) To what extent does this video make you think about your skills, abilities, and talents?. Scores from the two appearance-focussed questions (questions 1 and 2) were averaged, as we are focussed on the degree to which each video elicited appearance-focussed thoughts, regardless of the direction of these thoughts. To create an index of state self-objectification, scores from the skills/abilities question were subtracted from the averaged appearance-focussed scores. State self-objectification ranged from −4–4 with positive values indicating that participants focused on their appearance to a greater degree than their skills or abilities and negative scores indicating the reverse. We conducted a principle components analysis including all three items which found a single factor accounting for a large proportion of the cumulative variance (λ = 0.57). Additional information about this measure, including both theoretical and statistical considerations associated with this scale, is reported in supplementary materials (S1 Text).

### Analyses

To examine associations between trait/state self-objectification and mood when viewing appearance-focussed social media videos, we constructed five cross-classified multilevel regression models in which ratings were nested within two crossed factors (i.e., participants and video stimuli). To compare models, differences in fit between a model and its subsequent model were calculated and then compared to a chi-square distribution of significance [25]. Summaries of model fit across all nested models constructed for this study can be found in Supplementary Materials (S1 Table, S2 Table). All models showed adequate performance with respect to model assumptions.

Accounting for variance at both the subject- and video-level (as opposed to just the subject- or video-level) was the best fit for the data (S1 Table; *p*-values<0.05), suggesting that running cross-classified models would be most appropriate to assess the data. Per Nezlek [26], trait self-objectification was grand-mean centered, and state self-objectification was participant-mean (i.e., group-mean) centered across video categories before analyses. To account for multiple comparisons, we used the Benjamini-Hochberg false discovery rate procedure [27]. All reported *p*-values, both in the main text and supplementary materials, were adjusted using this procedure.

Our first set of models assessed whether trait self-objectification and video category interact in predicting ratings of state self-objectification after watching each video. General positive videos were used as the reference category to test our first hypothesis as those videos were not expected to impact self-objectification. We constructed the models hierarchically (beginning with an unconditional random-intercepts model) and found that the final model (S2 Table) including an interaction between trait self-objectification and video category fit the data best (*p*-values<0.001).

Next we ran four sets of models that assessed the interaction between trait self-objectification and video category on positive mood (1) and negative mood (2) as well as an interaction between state self-objectification and video category on positive mood (3) and negative mood (4). In these models, the neutral category was the reference as it was not expected to have a strong impact on mood. Again, we constructed models hierarchically. For Hypothesis 2, our final model assessing an interaction between video category and trait self-objectification on negative mood fit the data best (S3 Table), however, when considering state self-objectification, including this interaction term did not present any meaningful improvement in fit (∆_*D*_ < 10; S4 Table). In contrast when looking at positive mood (Hypothesis 3), adding trait self-objectification as a predictor did not meaningfully improve model fit (∆_*D*_ < 10; S5 Table) while for state self-objectification we found that the final model including an interaction between video category and state self-objectification fit the data best (S6 Table). Analyses were conducted in R 4.3.2 [28]. The complete deidentified dataset and analysis script are available on the study’s OSF page. The threshold for significance for results below is *p* < 0.05.

## Results

### State self-objectification (H1)

**Fig 1:**
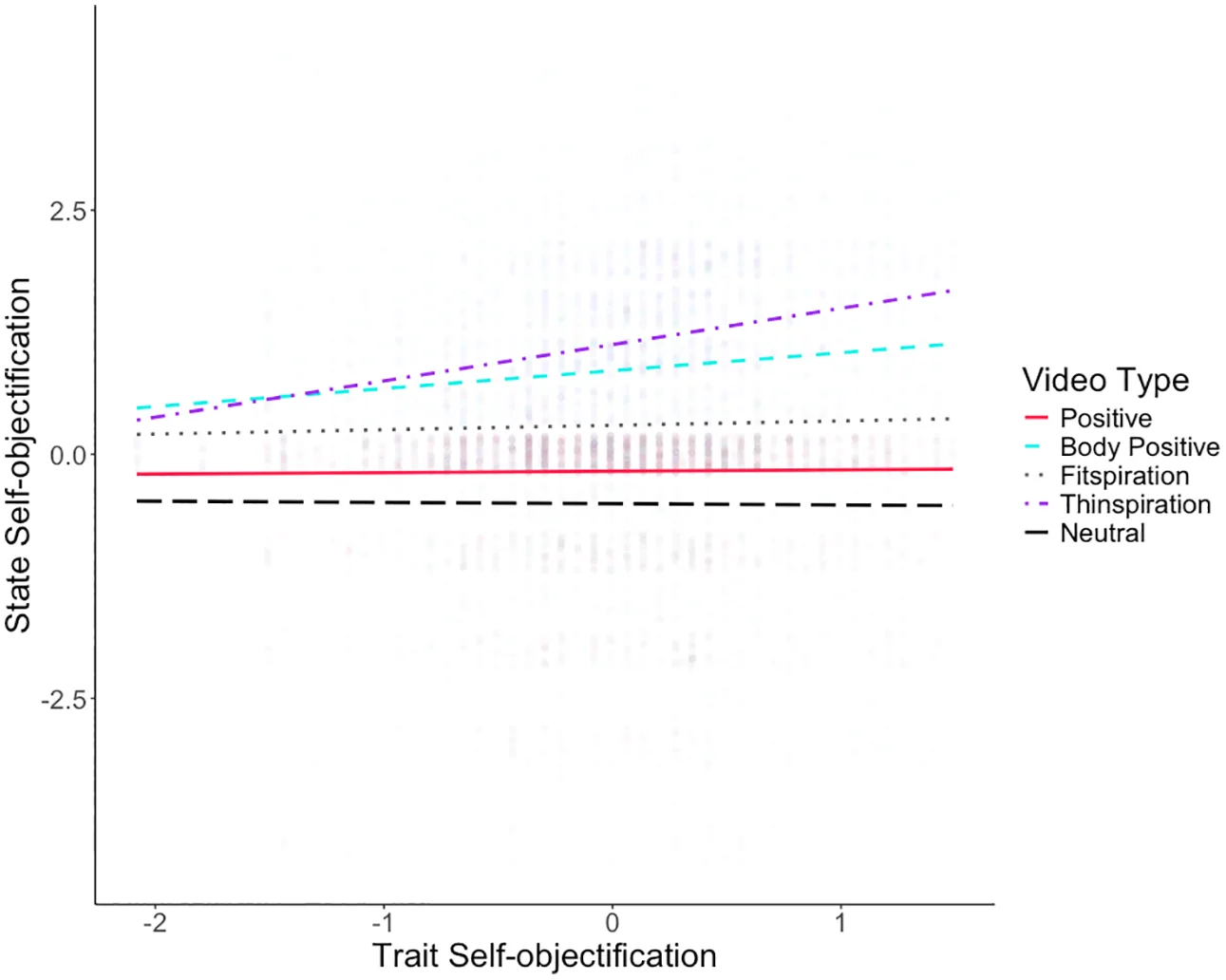
Associations between trait and state self-objectification across videos.

Compared to positive videos, state self-objectification was highest when viewing thinspiration videos (*B* = 1.24, *p* < 0.001) followed by body positive (*B* = 0.98, *p* < 0.001), and fitspiration (*B* = 0.43, *p* < 0.001). State self-objectification was lower when viewing the neutral videos (*B* = −0.34, *p* < 0.001) compared to positive videos. Trait self-objectification did not have a significant moderating effect on state self-objectification when watching non-appearance focussed videos (*p*-values>0.44). Somewhat consistent with hypotheses, trait self-objectification significantly moderated the effect of video category on state self-objectification (Fig 1) when viewing thinspiration (*B* = 0.41, *p* < 0.001) and body positive content (*B* = 0.18, *p* = 0.008) but not fitspiration (*B* = 0.08, *p* = 0.10). Relationships between these results and additional predictors (age and body size) are reported in Supplementary Materials (S3 Text). Neither age nor body size significantly predicted state self-objectification in the present sample, which was comprised of young, generally average-weight women.

### Negative Mood (H2A and H2B)

**Fig 2:**
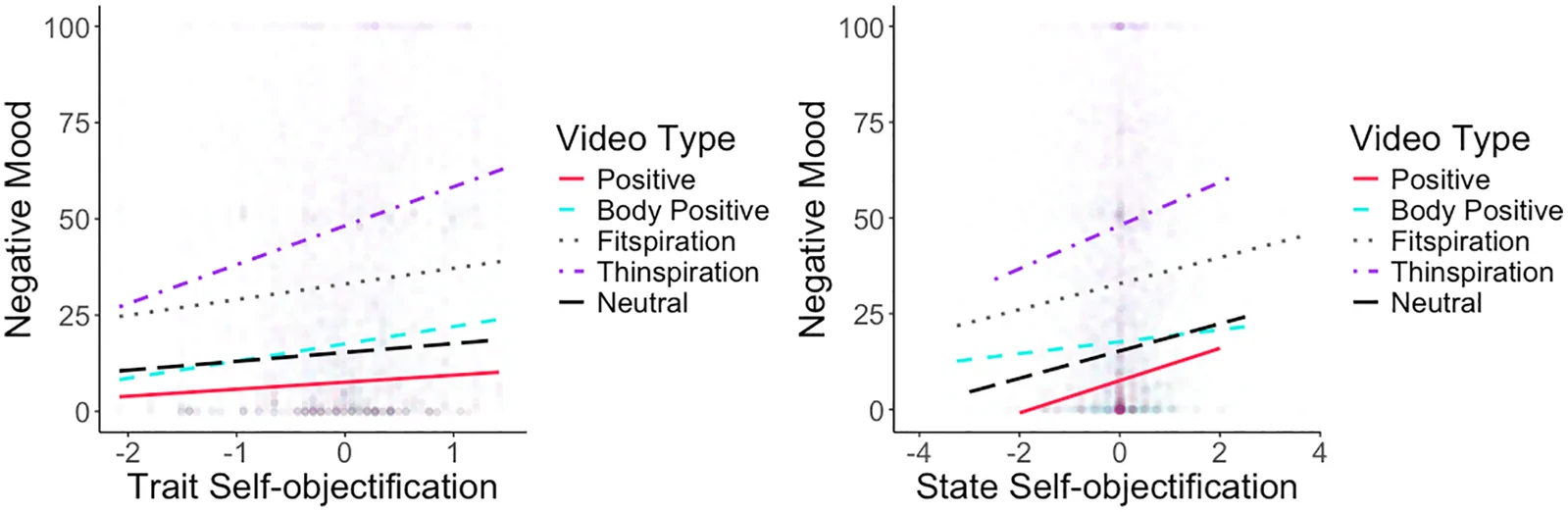
Associations between self-objectification and negative mood across videos. (A) Interaction between trait self-objectification and video category on negative mood. (B) Interaction between state self-objectification and video category on negative mood.

Across both models assessing negative mood, in comparison to neutral videos, viewing fitspiration and thinspiration was significantly associated with increases in negative mood (*p*-values<0.001, S3, S4), supporting our second hypothesis. Additionally, when compared to neutral videos, viewing positive videos was significantly associated with decreases in negative mood (*p*-values<0.001; S3, S4). Somewhat in support of H2A, the positive association between negative mood and thinspiration but not fitspiration was stronger for individuals higher in trait self-objectification (*B =* 7.49, *p* < 0.001; Fig 2A). Opposing H2B, state self-objectification was not a significant moderator in associations between thinspiration or fitspiration and negative mood (Fig 2B).

### Positive Mood (H3A and H3B)

**Fig 3:**
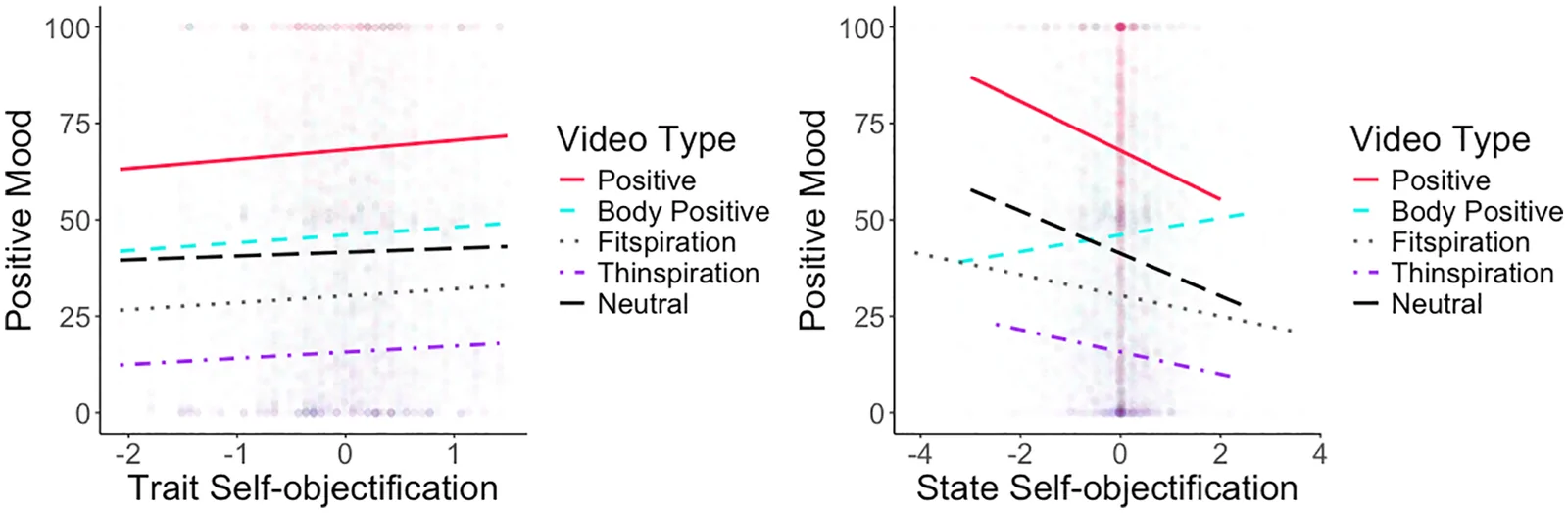
Associations between self-objectification and positive mood across videos. (A) Interaction between trait self-objectification and video category on positive mood. (B) Interaction between state self-objectification and video category on positive mood.

Compared to neutral videos, viewing non-appearance focussed positive videos and body positive videos were associated with increases in positive mood across our models (*p*-values<0.001; S5, S6). Additionally, viewing thinspiration and fitspiration was associated with decreases in positive mood (*p-*values<0.001; S5, S6), when compared to neutral videos. Contrary to H3A, trait self-objectification did not significantly predict positive mood across content categories (Fig 3A). However, we found support for H3B such greater state self-objectification when viewing body positive videos strengthened its effect on increasing positive mood (*B* = 6.49*, p* < 0.001; Fig 3B).

## Discussion

Viewing all forms of appearance-focussed social media (i.e., thinspiration, fitspiration, and body positive content) was associated with higher state self-objectification compared to viewing non body-focussed content. Consistent with previous literature, we found that higher levels of trait self-objectification was associated with greater state self-objectification after viewing certain forms appearance-focussed social media content [20], providing evidence for the circle of objectification theory [22]. However, trait self-objectification was only found to moderate the impact of appearance-focussed social media content on state self-objectification when viewing thinspiration and body positive content, but not fitspiration. While viewing fitspiration was independently associated with increases in state self-objectification, trait self-objectification did not play a significant role in this association. One possibility for this surprising result is that unlike viewing thinspiration and body positive content, fitspiration content did not trigger strong appearance-focused comparisons – a critical component in the circle of objectification theory [22]. Fitspiration, unlike thinspiration and body positive content, often includes instructions and tips for building fitness skills, which may split viewers’ focus between their ability and appearance. Thus, the combined ability and appearance focus in fitspiration may have reduced appearance-focussed comparisons among those high in trait self-objectification, leading to state self-objectification scores that were more similar to their peers with an average or low tendency to self-objectify.

We also predicted that participants higher in trait self-objectification would be more vulnerable to the negative effects of thinspiration and fitspiration on mood but simultaneously more likely to experience positive mood effects of body positive content. Somewhat consistent with hypotheses, higher levels of trait self-objectification strengthened the effect of viewing thinspiration but not fitspiration on negative mood. Additionally, counter to our hypotheses, trait self-objectification did not enhance positive mood across any content category. These results in combination with our finding that viewing thinspiration leads to greater state self-objectification suggest that increased self-objectification is not only an outcome of viewing thinspiration but also a vulnerability factor for its effects on negative mood. Given the widespread use of algorithms on social media and their impact on decision-making [29], future work should investigate how individual-level factors like trait self-objectification may further shape users’ experiences in these algorithmic systems. These findings highlight the negative outcomes associated with self-objectification [5]. Moreover, this work emphasizes the sociocultural pressures [30] involved in viewing idealized appearance-focussed (i.e., thinspiration) social media and their impact on self-objectification [31]. Future work should explore strategies for mitigating the harms of thinspiration, especially for those with a high tendency to self-objectify.

Though self-objectification can be a vulnerability factor for negative outcomes, recent research examining body positive content suggests that self-objectification may coexist with positive feelings [11]. Thus, we sought to examine the impact of different categories of appearance-focussed videos on self-objectification and mood. We predicted and found that while all forms of appearance-focussed social media content were associated with differences in state self-objectification, these momentary thoughts were only related to mood when viewing body positive content. Consistent with hypotheses, we found that experiencing greater state self-objectification strengthened the association between viewing body positive content and positive mood. Indeed, although we found that body positive content enhances individuals’ state self-objectification, the link between these thoughts and mood levels in our study provide evidence that self-objectifying thoughts can be positive in nature [32]. Thus, our results suggest that though appearance-focussed social media content can increase state self-objectification, these thoughts can be associated with positive mood outcomes when the content involves messages of body positivity.

Although the current study highlights the real-time effects of various social media content on mood and self-objectification, it cannot determine longitudinal effects. Thus, it is unclear how momentary increases in self-objectification when viewing appearance-focussed social media may impact mood over time. Additionally, although mood differences and state levels of self-objectification were assessed with reference to each video, we did not conduct a baseline mood or state self-objectification measure before the task began as the purpose of our study was to determine the relationship between content category, mood and state self-objectification. To extend this work, future studies should assess baseline mood and state self-objectification to gain a better understanding of how engaging with social media content shifts people’s moods and self-objectification over time. In addition, future work might consider testing a larger sample size and including participants with a more diverse range of body sizes so that potential covariates, including body size and age, might be adequately examined.

The current study assessed associations between self-objectification (state and trait) and mood when engaging with various types of social media content. Results indicate that although appearance-focussed social media content gives rise to self-objectification, this is not always negative. Indeed, when viewing body positive content, increased state levels of self-objectification strengthened the association between viewing body positive content and mood. Additionally, this study highlights the relationship between trait self-objectification and both state self-objectification and mood when viewing appearance-focussed content. Our findings suggest that women and feminine-identifying individuals with a greater propensity to self-objectify experience a greater self-objectification after viewing thinspiration and body positive content, and are more sensitive to thinspiration-induced negative mood effects. Future research assessing both the positive and negative outcomes of self-objectification would enhance knowledge in this field and inform interventions targeting body image, mood and the impact of digital media.

## Supporting information

S1 Text Stimulus Selection; State Self-Objectification Measure Analysis. (DOCX)

S2 Text Model Summaries. (DOCX)

S1 Table A) Comparison of unconditional models for variance in state self-objectification, positive, and negative mood – full sample. *Note.* Estimates are unstandardized beta values. Unconditional Model 1 = assesses variance in DV across participants; Unconditional Model 2 = assesses variance in DV across each unique video; Unconditional Model 3 = assesses variance in DV across both participants and each unique video. PID = Participant. vid = video. SO=self-objectification. DV = dependent variable. ICC = intraclass correlation. *p < 0.05, **p < 0.01, ***p < 0.001. B) Comparison of unconditional models for positive, and negative mood – subset sample. *Note.* Data was subset to ensure all models were fit to the same sample, to account for missing values in state self-objectification scores. Estimates are unstandardized beta values. Unconditional Model 1 = assesses variance in DV across participants; Unconditional Model 2 = assesses variance in DV across each unique video; Unconditional Model 3 = assesses variance in DV across both participants and each unique video. DV = dependent variable. PID = Participant. vid = video. ICC = intraclass correlation. *p < 0.05, **p < 0.01, ***p < 0.001.
(DOCX)

S2 Table Final cross-classified model assessing the effect of video category and trait self-objectification on state self-objectification. SO=self-objectification. CI = confidence interval. ICC = Intraclass correlation. *p < 0.05, **p < 0.01, ***p < 0.001.
(DOCX)

S3 Table Final cross-classified model assessing the effect of video category and trait self-objectification on negative mood. SO=self-objectification. CI = confidence interval. ICC = intraclass correlation. *p < 0.05, **p < 0.01, ***p < 0.001.
(DOCX)

S4 Table Final cross-classified model assessing the effect of video category and state self-objectification on negative mood. SO=self-objectification. CI = confidence interval. ICC = intraclass correlation. *p < 0.05, **p < 0.01, ***p < 0.001.
(DOCX)

S5 Table Final cross-classified model assessing the effect of video category on positive mood. SO=self-objectification. CI = confidence interval. ICC = intraclass correlation. *p < 0.05, **p < 0.01, ***p < 0.001.
(DOCX)

S6 Table Final cross-classified model assessing the effect of video category and state self-objectification on positive mood. SO=self-objectification. CI = confidence interval. ICC = intraclass correlation. *p < 0.05, **p < 0.01, ***p < 0.001.
(DOCX)

S3 Text Analysis of Potential Covariates (Age and Body Size). (DOCX)

## References

[1] Calogero RM. Objectification Theory, Self-Objectification, and Body Image. Encyclopedia of Body Image and Human Appearance. Elsevier; 2012. pp. 574–80. 10.1016/B978-0-12-384925-0.00091-2

[2] Fredrickson BL, Roberts T-A. Objectification Theory: Toward Understanding Women’s Lived Experiences and Mental Health Risks. Psychology of Women Quarterly. 1997;21(2):173–206. doi: 10.1111/j.1471-6402.1997.tb00108.x

[3] Miner-Rubino K, Twenge JM, Fredrickson BL. Trait Self-Objectification in Women: Affective and Personality Correlates. J Res Personality. 2002;36(2):147–72. doi: 10.1006/jrpe.2001.2343

[4] Tiggemann M, Kuring JK. The role of body objectification in disordered eating and depressed mood. Br J Clin Psychol. 2004;43(Pt 3):299–311. doi: 10.1348/0144665031752925 15333234

[5] Tiggemann M, Williams E. The role of self-objectification in disordered eating, depressed mood, and sexual functioning among women: a comprehensive test of objectification theory. Psychology of Women Quarterly. 2012;36:66–75. doi: 10.1177/0361684311420250

[6] Kroon Van Diest AM, Perez M. Exploring the integration of thin-ideal internalization and self-objectification in the prevention of eating disorders. Body Image. 2013;10(1):16–25. doi: 10.1016/j.bodyim.2012.10.004 23182310

[7] Fardouly J, Diedrichs PC, Vartanian LR, Halliwell E. The Mediating Role of Appearance Comparisons in the Relationship Between Media Usage and Self-Objectification in Young Women. Psychology of Women Quarterly. 2015;39(4):447–57. doi: 10.1177/0361684315581841

[8] Garcia RL, Bingham S, Liu S. The effects of daily Instagram use on state self-objectification, well-being, and mood for young women. Psychology of Popular Media. 2022;11(4):423–34. doi: 10.1037/ppm0000350

[9] Ghaznavi J, Taylor LD. Bones, body parts, and sex appeal: An analysis of #thinspiration images on popular social media. Body Image. 2015;14:54–61. doi: 10.1016/j.bodyim.2015.03.006 25880783

[10] Bell BT, Talbot CV, Deighton-Smith N. Following up on #fitspiration: A comparative content analysis and thematic analysis of social media content aiming to inspire fitness from 2014 and 2021. Psychology of Popular Media. 2024;13(4):666–76. doi: 10.1037/ppm0000523

[11] Cohen R, Fardouly J, Newton-John T, Slater A. #BoPo on Instagram: An experimental investigation of the effects of viewing body positive content on young women’s mood and body image. New Media & Society. 2019;21(7):1546–64. doi: 10.1177/1461444819826530

[12] Gurtala JC, Fardouly J. Does medium matter? Investigating the impact of viewing ideal image or short-form video content on young women’s body image, mood, and self-objectification. Body Image. 2023;46:190–201. doi: 10.1016/j.bodyim.2023.06.005 37354877

[13] Xu Y. Fitspiration, related risks, and coping strategies: Recent trends and future directions. Psychology of Popular Media. 2024;13(4):748–54. doi: 10.1037/ppm0000491

[14] Barnes K, Newman E, Keenan G. A comparison of the impact of exposure to fit ideal and non-fit ideal body shapes in fitspiration imagery on women. Computers in Human Behavior. 2023;144:107728. doi: 10.1016/j.chb.2023.107728

[15] Slater A, Varsani N, Diedrichs PC. #fitspo or #loveyourself? The impact of fitspiration and self-compassion Instagram images on women’s body image, self-compassion, and mood. Body Image. 2017;22:87–96. doi: 10.1016/j.bodyim.2017.06.004 28689104

[16] Sastre A. Towards a radical body positive: Reading the online “body positive movement.”. Feminist Media Studies. 2014;14:929–43. doi: 10.1080/14680777.2014.883420

[17] Dhadly PK, Kinnear A, Bodell LP. #BoPo: Does viewing body positive TikTok content improve body satisfaction and mood?. Eat Behav. 2023;50:101747. doi: 10.1016/j.eatbeh.2023.101747 37263141

[18] Webb JB, Vinoski ER, Bonar AS, Davies AE, Etzel L. Fat is fashionable and fit: A comparative content analysis of Fatspiration and Health at Every Size® Instagram images. Body Image. 2017;22:53–64. doi: 10.1016/j.bodyim.2017.05.003 28624756

[19] Cohen R, Irwin L, Newton-John T, Slater A. #bodypositivity: A content analysis of body positive accounts on Instagram. Body Image. 2019;29:47–57. doi: 10.1016/j.bodyim.2019.02.007 30831334

[20] Tiggemann M, Boundy M. Effect of Environment and Appearance Compliment on College Women’s Self-Objectification, Mood, Body Shame, and Cognitive Performance. Psychology of Women Quarterly. 2008;32(4):399–405. doi: 10.1111/j.1471-6402.2008.00453.x

[21] McKinley NM, Hyde JS. The Objectified Body Consciousness Scale: Development and Validation. Psychology of Women Quarterly. 1996;20: 181–215. doi: 10.1111/j.1471-6402.1996.tb00467.x

[22] Lindner D, Tantleff-Dunn S, Jentsch F. Social Comparison and the ‘Circle of Objectification’. Sex Roles. 2012;67:222–35. doi: 10.1007/s11199-012-0175-x

[23] Monteiro D, Poulakis M. Effects of Cisnormative Beauty Standards on Transgender Women’s Perceptions and Expressions of Beauty. Midwest Social Sciences Journal. 2019;22(1). doi: 10.22543/2766-0796.1009

[24] Lindner D, Tantleff-Dunn S. The Development and Psychometric Evaluation of the Self-Objectification Beliefs and Behaviors Scale. Psychology of Women Quarterly. 2017;41(2):254–72. doi: 10.1177/0361684317692109

[25] Snijders TA, Bosker R. Multilevel analysis: An introduction to basic and advanced multilevel modeling. 2011.

[26] Nezlek JB. An Introduction to Multilevel Modeling for Social and Personality Psychology. Social & Personality Psych. 2008;2(2):842–60. doi: 10.1111/j.1751-9004.2007.00059.x

[27] Benjamini Y, Hochberg Y. Controlling the False Discovery Rate: A Practical and Powerful Approach to Multiple Testing. Journal of the Royal Statistical Society Series B: Statistical Methodology. 1995;57(1):289–300. doi: 10.1111/j.2517-6161.1995.tb02031.x

[28] R Core Team. R: A language and environment for statistical computing. Vienna, Austria: R Foundation for Statistical Computing. 2023.

[29] Jones SM, Heerey EA. Resilience and disempowerment in algorithmic systems. New Media & Society. 2026. doi: 10.1177/14614448261446591

[30] Liu Z, Fu M, Shi J, Hu Y, Gao X. The psychological mechanism of Self-objectification: the interaction between sociocultural pressures and the self-system. Front Psychol. 2025;16:1531222. doi: 10.3389/fpsyg.2025.1531222 41089645 PMC12517067

[31] Pritchard M, Button A. #Instabod versus #BoPo: An experimental study of the effects of viewing idealized versus body-positive content on collegiate males’ and females’ body satisfaction. Psychology of Popular Media. 2024;13(3):291–302. doi: 10.1037/ppm0000454

[32] Rodgers RF, Wertheim EH, Paxton SJ, Tylka TL, Harriger JA. #Bopo: Enhancing body image through body positive social media- evidence to date and research directions. Body Image. 2022;41:367–74. doi: 10.1016/j.bodyim.2022.03.008 35525155

