# Supplementary material for: The impact of appearance-focussed social media and self-objectification on mood
Source: PLoS One. 2026 Sep 9;21(9):e0355465. doi: 10.1371/journal.pone.0355465 (PMC13557393; doi:10.1371/journal.pone.0355465)
Supplement: Supplementary file 1 [file pone.0355465.s001.docx]

**Stimulus Selection**

To collect the videos, researchers used search terms such as #bopo, #fitness, #gym, #diy, #cuteanimal, etc. Researchers also used hashtags such as #th!nspo or #th1nspo which are created by users to act as a loophole to TikTok’s attempt to restrict searchers from content that might promote disordered eating. TikTok videos were also selected using the app’s “for you page” (FYP). FYPs are algorithmically curated feeds of personalized content constructed specifically for each user. The three researchers collecting videos were instructed to create new TikTok accounts to allow their FYPs to become personalized for the type of content desired for this study. Researchers collected and agreed upon 91 videos for the thinspiration category, 79 fitspiration, 83 body positive, 65 positive and 72 neutral for a total of 390 unique videos.

Importantly, the videos containing people included a diverse range of women/feminine-presenting individuals, including creators whose appearance might lead a viewer to infer that they were Asian, Black, Hispanic, Indian, Persian, White, etc. However, ethnicity/race is an extremely individualized variable. It is neither possible nor appropriate to make inferences about a person’s race/ethnicity based only on their appearance. Because such demographic information is not systematically available for TikTok content creators, we were unable to characterize the diversity of the visible people in the video sample in a valid and principled manner. We are therefore unable to describe this aspect of the stimuli with precision.

As a manipulation check, participants in the current study were asked to categorize each video they viewed. Specifically, participants were asked: “Please select and drag any categories you feel describe this video into the empty box on the right. In the box, please order your selection(s) from the most (top) to least applicable (bottom) if you choose more than one”. The categories participants were able to choose from are listed below:

- Category A: videos focussing on, valuing, and/or endorsing **thinness**
- Category B: videos focussing on, valuing, and/or endorsing a **fit physique**
- Category C: videos focussing on, valuing, and/or endorsing **body and/or fat acceptance**
- Category D: videos that may make one **feel good**
- Category E: videos that are uninteresting, boring, or neutral
- None of the above/Other

Videos that researchers categorized as thinspiration was identified as Category A 89.5% of the time, fitspiration was identified as Category B 91.9% of the time, body positivity was identified as Category C 91.8% of the time, non-appearance focussed positive videos were identified as Category D 86.1% of the time, and non-appearance focussed neutral videos were identified as Category E 64.8% of the time and Category D 44.8% of the time.

**State Self-Objectification Measure**

To operationalize state self-objectification, which combines both positive (things a person likes) and negative thoughts (things a person dislikes) about their body, contrasted with focus on skills/abilities we used the following rationale. First, from a theoretical perspective, the definition of self-objectification involves the prioritization of appearance over skills and/or traits (Fredrickson, et al., 1998; Liu, et al., 2025). Although it is often presumed to be negative, self-objectification can be associated with positive outcomes, such as short-term rises in self-esteem (Chen, et al., 2023; Grower & Ward, 2021). These effects may be particularly prominent with respect to social media (Cohen, et al., 2018). Thus, we thought it important to measure the negative aspects of self-objectification along with the positive to capture this construct in a nuanced way. Second, social media platforms are designed to make consumers experience positive feelings in addition to any negative feelings they might experience. Although body-focused content may indeed be self-objectifying in a negative way, such content must promote enough positive feeling to attract views, likes, and shares to be promoted by an algorithm. Finally, we note that in the emotion literature, evidence shows that both positively and negatively valenced emotions regularly co-occur and may be positively correlated (Larson, et al., 2017, Souders & Yu, 2025), meaning that both types of feelings may co-occur in the context of self-objectification as well. On that theoretical basis, we planned to measure this construct by asking participants to tell us about both the positive and negative feelings they experienced about their bodies, contrasted with focus on abilities.

From a statistical perspective, before we began our analysis, we examined the data to ensure that this plan was reasonable. We began by examining the correlation between our body-focused measures (like body; dislike body) with a Spearman’s rho, as the scale data are ordinal. Participants’ perceptions of the things they liked and disliked about their bodies was positively correlated (rho = .43, p < .001). Next, we asked how far apart the like-body and dislike-body ratings were from one another. To obtain this estimate, we calculated the average absolute value of the difference between the like/dislike ratings for each participant. The median and modal difference was 0.70 (mean = 0.75; SD = 0.46). Finally, to examine the relationship between the like/dislike body items and how people thought about their skills together as a short scale, we performed a PCA including all three variables. The PCA results suggested a single factor accounting for a large proportion of the cumulative variance (eigenvalue = 1.72; proportion of variance accounted for across the three items = .57). The absolute value of all factor loadings was high (like body: .82; dislike body = .80; skills focus = .64). Because the like and dislike body items are similarly rated and correlated, we averaged them to obtain a measure of body-focused attention. We then contrasted this measure with skill focus by subtracting this from the body focused rating to obtain our metric of the relative weighting of body versus skill focus. Together, these results suggest that our trio of items is a reasonable measure of state self-objectification.

**References**

Chen, S., van Tilburg, W. A., & Leman, P. J. (2023). Women's self-objectification and strategic self-presentation on social media. *Psychology of women quarterly*, *47*(2), 266-282.

Cohen, R., Newton-John, T., & Slater, A. (2018). ‘Selfie’-objectification: The role of selfies in self-objectification and disordered eating in young women. *Computers in Human Behavior*, *79*, 68-74.

Fredrickson, B., Roberts, T., Noll, S., Quinn, D., & Twenge, J. M. (1998). That swimsuit becomes you: sex differences in self-objectification, restrained eating, and math performance. *J. Pers. Soc. Psychol., 75*, 269–284.

Grower, P., & Ward, L. M. (2021). Differentiating contributions of self-objectification and self-sexualization to young women’s sexual agency. *Body image*, *38*, 63-71.

Larsen, J., Hershfield, H., Stastny, B., & Hester, N. (2017). On the relationship between positive and negative affect: Their correlation and their co-occurrence. *Emotion*, *17*(2), 323.

Liu, Z., Fu, M., Shi, J., Hu, Y., & Gao, X. (2025) The psychological mechanism of Self-objectification: the interaction between sociocultural pressures and the self-system. *Front. Psychol.* 16:1531222.

Souders, D.J., & Yu, Y. (2025). Beyond the bipolar: allowing satisfaction and dissatisfaction to coexist. *Front Psychol.* 16:1572220.
