# Supplementary material for: The impact of appearance-focussed social media and self-objectification on mood
Source: PLoS One. 2026 Sep 9;21(9):e0355465. doi: 10.1371/journal.pone.0355465 (PMC13557393; doi:10.1371/journal.pone.0355465)
Supplement: Supplementary file 2 [file pone.0355465.s002.docx]

**Model Summaries**

Below are fit indices (e.g., Akaike Information Criterion [AIC], Bayesian Information Criterion [BIC])  for each of the hieratically analyzed models in our sample. Difference in deviance statistics across models were compared to a chi-square distribution of significance (Snijders & Bosker, 2011).

Table 1. Comparison of unconditional models for variance in state self-objectification, positive, and negative mood – full sample.

| model | AIC | BIC | - Log Likelihood | Deviance |
| --- | --- | --- | --- | --- |
| Unconditional Model 1A  Outcome: State self-objectification  Variance: Participant-level | 19589.82 | 19610.12 | -9791.91 | 19583.82 |
| Unconditional Model 2A  Outcome: State self-objectification  Variance: Video-level | 17589.95 | 17610.26 | -8791.98 | 17583.95 |
| Unconditional Model 3A  Outcome: State self-objectification  Variance: Participant-level, video-level | 17308.62 | 17335.69 | -8650.31 | 17300.62 |
| Unconditional Model 1B  Outcome: Positive mood  Variance: Participant-level | 39108.72 | 39127.70 | -19551.36 | 39102.72 |
| Unconditional Model 2B  Outcome: Positive mood  Variance: Video-level | 39042.55 | 39061.54 | -19518.28 | 39036.55 |
| Unconditional Model 3B  Outcome: Positive mood  Variance: Participant-level, video-level | 38242.46 | 38267.78 | -19117.23 | 38234.46 |
| Unconditional Model 1C  Outcome: Negative mood  Variance: Participant-level | 50400.82 | 50420.51 | -25197.41 | 50394.82 |
| Unconditional Model 2C  Outcome: Negative mood  Variance: Video-level | 49577.16 | 49596.851 | -24785.58 | 49571.16 |
| Unconditional Model 3C  Outcome: Negative mood  Variance: Participant-level, video-level | 48650.04 | 48676.28 | -24321.02 | 48642.04 |

Table 2. Comparison of unconditional models for positive, and negative mood – subset sample.

| model | AIC | BIC | - Log Likelihood | Deviance |
| --- | --- | --- | --- | --- |
| Unconditional Model 1A  Outcome: Positive mood  Variance: Participant-level | 38174.44 | 38193.35 | -19084.22 | 38168.44 |
| Unconditional Model 2A  Outcome: Positive mood  Variance: Video-level | 38133.38 | 38152.29 | -19063.69 | 38127.38 |
| Unconditional Model 3A  Outcome: Positive mood  Variance: Participant-level, video-level | 37362.47 | 37387.69 | -18677.23 | 37354.47 |
| Unconditional Model 1B  Outcome: Negative mood  Variance: Participant-level | 49350.12 | 49369.75 | -24672.06 | 49344.12 |
| Unconditional Model 2B  Outcome: Negative mood  Variance: Video-level | 48612.60 | 48632.23 | -24303.30 | 48606.61 |
| Unconditional Model 3B  Outcome: Negative mood  Variance: Participant-level, video-level | 47669.47 | 47695.64 | -23830.74 | 47661.48 |

Table 3. Assessing the interaction between trait self-objectification and video type on state self-objectification.

| **model** | **AIC** | **BIC** | **- Log Likelihood** | **Deviance** |
| --- | --- | --- | --- | --- |
| **Model 1**  Addition of level 1 predictor (video type) | 16805.49 | 16859.63 | -8394.74 | 16789.49 |
| **Model 2**  Addition of level 2 (trait self-objectification) | 16792.51 | 16853.42 | -8387.26 | 16774.51 |
| **Model 3**  Add interaction (video type x trait self-objectification | 16725.82 | 16813.79 | -8349.91 | 16699.82 |

Table 4. Assessing the interaction between trait self-objectification and video type on negative mood.

| **model** | **AIC** | **BIC** | **- Log Likelihood** | **Deviance** |
| --- | --- | --- | --- | --- |
| **Model 1**  Addition of level 1 predictor (video type) | 37743.74 | 37794.37 | -18863.87 | 37727.74 |
| **Model 2**  Addition of level 2 predictor (trait self-objectification) | 37730.16 | 37787.11 | -18856.08 | 37712.16 |
| **Model 3**  Add interaction (video type x trait self-objectification | 37699.84 | 37782.10 | -18836.92 | 37673.84 |

Table 5. Assessing the interaction between state self-objectification and video type on negative mood.

| model | AIC | BIC | - Log Likelihood | Deviance |
| --- | --- | --- | --- | --- |
| Model 1  Addition of level 1 predictor (video type) | 36864.57 | 36915.01 | -18424.29 | 36848.57 |
| Model 2  Addition of level 2 predictor (state self-objectification) | 36827.56 | 36884.30 | -18404.78 | 36809.56 |
| Model 3  Add interaction (video type x state self-objectification | 36829.09 | 36911.05 | -18401.54 | 36803.09 |

Table 6. Assessing the interaction between trait self-objectification and video type on positive mood.

| model | AIC | BIC | - Log Likelihood | Deviance |
| --- | --- | --- | --- | --- |
| Model 1  Addition of level 1 predictor (video type) | 48064.09 | 48116.59 | -24024.05 | 48048.09 |
| Model 2  Addition of level 2 predictor (trait self-objectification) | 48064.40 | 48123.46 | -24023.20 | 48046.40 |
| Model 3  Add interaction (video type x trait self-objectification) | 48071.19 | 48156.49 | -24022.59 | 48045.19 |

Table 7. Assessing the interaction between state self-objectification and video type on positive mood.

| model | AIC | BIC | - Log Likelihood | Deviance |
| --- | --- | --- | --- | --- |
| Model 1  Addition of level 1 predictor (video type) | 47096.75 | 47149.09 | -23540.38 | 47080.75 |
| Model 2  Addition of level 2 predictor (state self-objectification) | 47084.53 | 47143.42 | -23533.27 | 47066.53 |
| Model 3  Add interaction (video type x state self-objectification) | 47061.82 | 47146.87 | -23517.91 | 47035.82 |
