# Supplementary material for: The impact of appearance-focussed social media and self-objectification on mood
Source: PLoS One. 2026 Sep 9;21(9):e0355465. doi: 10.1371/journal.pone.0355465 (PMC13557393; doi:10.1371/journal.pone.0355465)
Supplement: Supplementary file 3 [file pone.0355465.s003.docx]

S1 Table A. Comparison of unconditional models for variance in state self-objectification, positive, and negative mood – full sample.

|  | Unconditional Model 1 | | | Unconditional Model 2 | | | Unconditional Model 3 | | |
| --- | --- | --- | --- | --- | --- | --- | --- | --- | --- |
| Outcome | State SO | Negative Mood | Positive Mood | State SO | Negative Mood | Positive Mood | State SO | Negative Mood | Positive Mood |
| **Fixed Effects** | *Estimates* | *Estimates* | *Estimates* | *Estimates* | *Estimates* | *Estimates* | *Estimates* | *Estimates* | *Estimates* |
| Intercept  β(SE) | 0.32  (0.02)*** | 31.23  (0.94)*** | 43.79  (0.85)*** | 0.38  (0.04)*** | 26.42  (0.90)*** | 38.90  (1.01)*** | 0.38  (0.04)*** | 27.31  (1.15)*** | 38.87  (1.23)*** |
| **Random Effects** | | | | | | | | | |
| Residual | 1.19 | 655.63 | 817.11 | 0.78 | 632.15 | 658.97 | 0.70 | 444.73 | 482.92 |
| Group-level | 0.06 _PID_ | 227.24 _PID_ | 177.65 _PID_ | 0.48 _vid_ | 244.28 _vid_ | 339.83 _vid_ | 0.48 _vid_ | 221.17 _vid_ | 337.36 _vid_ |
|  |  |  |  |  |  |  | 0.08 _PID_ | 197.06 _PID_ | 173.62 _PID_ |
| ICC | 0.05 | 0.26 | 0.18 | 0.38 | 0.28 | 0.34 | 0.44 | 0.48 | 0.51 |
| N | 323 _PID_ | 323 _PID_ | 323 _PID_ | 386 _vid_ | 386 _vid_ | 386 _vid_ | 323 _PID_ | 323 _PID_ | 323 _PID_ |
|  |  |  |  |  |  |  | 386 _vid_ | 386 _vid_ | 386 _vid_ |
| **Model Summary** |  |  |  |  |  |  |  |  |  |
| Observations | 6421 | 4137 | 5230 | 6421 | 4137 | 5230 | 6421 | 4137 | 5230 |
| Marginal R^2^ / Conditional R^2^ | 0.000 / 0.048 | 0.000 / 0.257 | 0.000 / 0.179 | 0.000 / 0.378 | 0.000 / 0.279 | 0.000 / 0.340 | 0.000 / 0.442 | 0.000 / 0.485 | 0.000 / 0.514 |

*Note.* Estimates are unstandardized beta values. Unconditional Model 1=assesses variance in DV across participants; Unconditional Model 2=assesses variance in DV across each unique video; Unconditional Model 3=assesses variance in DV across both participants and each unique video. PID=Participant. vid=video. SO=self-objectification. DV=dependent variable. ICC=intraclass correlation. **p*<0.05, ***p*<0.01, ****p*<0.001.

S1 Table B. Comparison of unconditional models for positive, and negative mood – subset sample.

|  | Unconditional Model 1 | | Unconditional Model 2 | | Unconditional Model 3 | |
| --- | --- | --- | --- | --- | --- | --- |
| Outcome | Negative Mood | Positive Mood | Negative Mood | Positive Mood | Negative Mood | Positive Mood |
| **Fixed Effects** | *Estimates* | *Estimates* | *Estimates* | *Estimates* | *Estimates* | *Estimates* |
| Intercept  β(SE) | 31.02(0.93)*** | 43.75(0.86)*** | 26.38(0.89)*** | 38.90(1.01)*** | 27.27(1.15)*** | 38.97(1.23)*** |
| **Random Effects** | | | | | | |
| Residual | 649.43 | 803.05 | 630.32 | 658.65 | 443.82 | 477.88 |
| Group-level | 222.51 _PID_ | 187.03 _PID_ | 237.29 _vid_ | 335.90 _vid_ | 216.52 _vid_ | 330.34 _vid_ |
|  |  |  |  |  | 195.69 _PID_ | 178.28 _PID_ |
| ICC | 0.26 | 0.19 | 0.27 | 0.34 | 0.48 | 0.52 |
| N | 323 _PID_ | 323 _PID_ | 386 _vid_ | 386 _vid_ | 323 _PID_ | 323 _PID_ |
|  |  |  |  |  | 386 _vid_ | 386 _vid_ |
| **Model Summary** |  |  |  |  |  |  |
| Observations | 4042 | 5128 | 4042 | 5128 | 4042 | 5128 |
| Marginal R^2^ / Conditional R^2^ | 0.000 / 0.255 | 0.000 / 0.189 | 0.000 / 0.273 | 0.000 / 0.338 | 0.000 / 0.482 | 0.000 / 0.516 |

*Note.* Data was subset to ensure all models were fit to the same sample, to account for missing values in state self-objectification scores. Estimates are unstandardized beta values. Unconditional Model 1=assesses variance in DV across participants; Unconditional Model 2=assesses variance in DV across each unique video; Unconditional Model 3=assesses variance in DV across both participants and each unique video. DV=dependent variable. PID=Participant. vid=video. ICC=intraclass correlation. **p*<0.05, ***p*<0.01, ****p*<0.001.
