# Supplementary material for: The impact of appearance-focussed social media and self-objectification on mood
Source: PLoS One. 2026 Sep 9;21(9):e0355465. doi: 10.1371/journal.pone.0355465 (PMC13557393; doi:10.1371/journal.pone.0355465)
Supplement: Supplementary file 4 [file pone.0355465.s004.docx]

S2 Table. Final cross-classified model assessing the effect of video category and trait self-objectification on state self-objectification.

|  | | *Estimates* | *CI* |
| --- | --- | --- | --- |
| Fixed Effects | |  |  |
| Intercept | | -0.17*** | -0.27 – -0.08 |
| Video Category: | |  | |
|  | Neutral | -0.34*** | -0.46 – -0.22 |
|  | Body Positive | 1.03*** | 0.91 – 1.15 |
|  | Fitspiration | 0.47*** | 0.35 – 0.59 |
|  | Thinspiration | 1.30*** | 1.18 – 1.41 |
| Trait SO | | -0.01 | -0.08 – 0.10 |
| Trait SO x Video Category | |  | |
|  | Trait SO x Neutral | -0.05 | -0.16 – 0.05 |
|  | Trait SO x Body Positive | 0.17** | 0.06 – 0.27 |
|  | Trait SO x Fitspiration | 0.05 | -0.06 – 0.15 |
|  | Trait SO x Thinspiration | 0.36*** | 0.26 – 0.47 |
| Random Effects | |  | |
| Residual | | 0.70 | |
| Participant-level | | 0.07 | |
| Video-level | | 0.10 | |
| ICC | | 0.20 | |
| Model Summary | |  | |
| Observations | | 6421 | |
| Marginal R^2^/Conditional R^2^ | | 0.31/0.45 | |

*Note.* SO=self-objectification. CI=confidence interval. ICC=intraclass correlation.**p*<0.05, ***p*<0.01, ****p*<0.001.
