# Supplementary material for: The impact of appearance-focussed social media and self-objectification on mood
Source: PLoS One. 2026 Sep 9;21(9):e0355465. doi: 10.1371/journal.pone.0355465 (PMC13557393; doi:10.1371/journal.pone.0355465)
Supplement: Supplementary file 5 [file pone.0355465.s005.docx]

S3 Table. Final cross-classified model assessing the effect of video category and trait self-objectification on negative mood

|  | | *Estimates* | *CI* |
| --- | --- | --- | --- |
| Fixed Effects | |  |  |
| Intercept | | 16.13*** | 13.51 – 18.75 |
| Trait SO | | 2.60 | -0.99 – 6.20 |
| Video Category: | |  | |
|  | Positive | -5.78*** | -8.93 – -2.62 |
|  | Body Positive | 2.03 | -0.85 – 4.91 |
|  | Fitspiration | 16.93*** | 14.15 – 19.72 |
|  | Thinspiration | 31.85*** | 29.14 – 34.56 |
| Video Category x Trait SO | |  | |
|  | Trait SO x Positive | -1.66 | -5.51 – 2.20 |
|  | Trait SO x Body Positive | 1.63 | -1.90 – 5.15 |
|  | Trait SO x Fitspiration | 0.96 | -2.40 – 4.32 |
|  | Trait SO x Thinspiration | 7.49*** | 4.18 – 10.81 |
| Random Effects | |  | |
| Residual | | 438.00 | |
| Participant-level | | 180.75 | |
| Video-level | | 29.27 | |
| ICC | | 0.32 | |
| Model Summary | |  | |
| Observations | | 4137 | |
| Marginal R^2^/Conditional R^2^ | | 0.25/0.49 | |

*Note.* SO=self-objectification. CI=confidence interval. ICC=intraclass correlation. **p*<0.05, ***p*<0.01, ****p*<0.001.
