# Supplementary material for: The impact of appearance-focussed social media and self-objectification on mood
Source: PLoS One. 2026 Sep 9;21(9):e0355465. doi: 10.1371/journal.pone.0355465 (PMC13557393; doi:10.1371/journal.pone.0355465)
Supplement: Supplementary file 6 [file pone.0355465.s006.docx]

S4 Table. Final cross-classified model assessing the effect of video category and state self-objectification on negative mood

|  | | *Estimates* | *CI* |
| --- | --- | --- | --- |
| Fixed Effects | |  |  |
| Intercept | | 16.13*** | 13.52 – 18.74 |
| State SO | | 3.19*** | 2.19 – 4.19 |
| Video Category: | |  | |
|  | Positive | -5.60*** | -8.71 – -2.50 |
|  | Body Positive | 2.07 | -0.75 – 4.89 |
|  | Fitspiration | 16.75*** | 14.03 – 19.47 |
|  | Thinspiration | 31.88*** | 29.23 – 34.52 |
| Random Effects | |  | |
| Residual | | 428.90 | |
| Participant-level | | 189.80 | |
| Video-level | | 24.30 | |
| ICC | | 0.33 | |
| Model Summary | |  | |
| Observations | | 5128 | |
| Marginal R^2^/Conditional R^2^ | | 0.23/0.49 | |

*Note.* SO=self-objectification. CI=confidence interval. ICC=intraclass correlation. **p*<0.05, ***p*<0.01, ****p*<0.001.
