# Supplementary material for: The impact of appearance-focussed social media and self-objectification on mood
Source: PLoS One. 2026 Sep 9;21(9):e0355465. doi: 10.1371/journal.pone.0355465 (PMC13557393; doi:10.1371/journal.pone.0355465)
Supplement: Supplementary file 7 [file pone.0355465.s007.docx]

S5 Table. Final cross-classified model assessing the effect of video category on positive mood

|  | | *Estimates* | *CI* |
| --- | --- | --- | --- |
| Fixed Effects | |  |  |
| Intercept | | 41.22*** | 38.71 – 43.73 |
| Video Category: | |  | |
|  | Positive | 26.98*** | 24.10 – 29.85 |
|  | Body Positive | 4.75*** | 1.95 – 7.55 |
|  | Fitspiration | -11.16*** | -14.03 – -8.29 |
|  | Thinspiration | -25.34*** | -28.28 – -22.41 |
| Random Effects | |  | |
| Residual | | 481.44 | |
| Participant-level | | 174.36 | |
| Video-level | | 42.64 | |
| ICC | | 0.31 | |
| Model Summary | |  | |
| Observations | | 5230 | |
| Marginal R^2^/Conditional R^2^ | | 0.30/0.51 | |

*Note.* CI=confidence interval. ICC=intraclass correlation. **p*<0.05, ***p*<0.01, ****p*<0.001.
