# Supplementary material for: The impact of appearance-focussed social media and self-objectification on mood
Source: PLoS One. 2026 Sep 9;21(9):e0355465. doi: 10.1371/journal.pone.0355465 (PMC13557393; doi:10.1371/journal.pone.0355465)
Supplement: Supplementary file 8 [file pone.0355465.s008.docx]

S6 Table. Final cross-classified model assessing the effect of video category and state self-objectification on positive mood

|  | | *Estimates* | *CI* |
| --- | --- | --- | --- |
| Fixed Effects | |  |  |
| Intercept | | 40.97*** | 38.46 – 43.47 |
| State SO | | -4.44*** | -6.49 – -2.38 |
| Video Category: | |  | |
|  | Positive | 26.96*** | 24.12 – 29.81 |
|  | Body Positive | 5.04*** | 2.27 – 7.81 |
|  | Fitspiration | -10.74*** | -13.59 – -7.89 |
|  | Thinspiration | -24.80*** | -27.72 – -21.89 |
| Video Category x State SO | |  | |
|  | State SO x Positive | -1.09 | -4.89 – 2.71 |
|  | State SO x Body Positive | 6.49*** | 3.78 – 9.19 |
|  | State SO x Fitspiration | 2.26 | -0.40 – 4.91 |
|  | State SO x Thinspiration | 1.56 | -1.87 – 5.00 |
| Random Effects | |  | |
| Residual | | 473.27 | |
| Participant-level | | 178.32 | |
| Video-level | | 40.72 | |
| ICC | | 0.32 | |
| Model Summary | |  | |
| Observations | | 5128 | |
| Marginal R^2^/Conditional R^2^ | | 0.29/0.52 | |

*Note.* SO=self-objectification. CI=confidence interval. ICC=intraclass correlation. **p*<0.05, ***p*<0.01, ****p*<0.001.
