# Supplementary material for: The impact of appearance-focussed social media and self-objectification on mood
Source: PLoS One. 2026 Sep 9;21(9):e0355465. doi: 10.1371/journal.pone.0355465 (PMC13557393; doi:10.1371/journal.pone.0355465)
Supplement: Supplementary file 9 [file pone.0355465.s009.docx]

**Additional Covariates**

**Body size:**

Participants reported their body size using a categorical response variable with categories ranging from very underweight to very overweight. The table below shows the proportion of participants responding in each category. As this table shows, over 82% of participants classified themselves as average or near average, meaning there is little variability across the sample.

**Table:** Percent of participants reporting membership in each body-size category.

| Not Reported | Very Underweight | Underweight | Slightly Underweight | Average weight | Slightly Overweight | Overweight | Very Overweight |
| --- | --- | --- | --- | --- | --- | --- | --- |
| 9.90% | 0.80% | 1.26% | 12.80% | 51.70% | 17.91% | 5.12% | 0.50% |

We ran the model with body size as categorical predictor (excluding the participants who chose not to report). None of the individual body-size categories significantly predicted state self-objectification as a main effect (uncorrected p-values > .07), a finding we expected due to the inclusion of content categories that are not body-associated/self-objectifying. When we added in the self-objectification x video-type interaction, where we might reasonably expect body-size to play a role, this led to unacceptably inflated VIF statistics (body size: 135.54; body size x video type: 284.82). These data suggest significant multicollinearity across these predictors, so much so that model results are not interpretable.

**Age:**

Previous research suggests that feelings of self-objectification decline with age (1,2). The current sample was mainly comprised of young adults (mean age = 21.53 years; SD = 3.29; interquartile range = 19 to 23). To examine whether age-related effects were associated with state self-objectification in the present sample, we ran our models with mean-centered age as a predictor. Age did not significantly predict state self-objectification (standardized coefficient: < 0.01, uncorrected p=.368). Including the interaction between age and video-type showed a significant inflation in state self-objectification for fitspiration videos only as age increased (standardized coefficient: 0.12, uncorrected p < .001).

One plausible explanation for both of these results is that in the present sample of young, near-average weight women, these factors are less influential than previous reports suggest, perhaps because members of this demographic experience such content so regularly that it has become normalized. With respect to age, we note that our sample is quite homogenous in terms of age (young adults), whereas previous work finding age effects in self-objectification (1,2) has included samples of older women (typically over 40 years old.

**References**

1.	Grippo KP, Hill MS. Self-objectification, habitual body monitoring, and body dissatisfaction in older European American women: Exploring age and feminism as moderators. Body Image. 2008 Jun 1;5(2):173–82. doi:10.1016/j.bodyim.2007.11.003

2.	Sherman AM, Tran S, Sy J. Objectification and body esteem: age group patterns in women’s psychological functioning. Aging & Mental Health. 2024 Apr 2;28(4):706–16. doi:10.1080/13607863.2023.2273338 PubMed PMID: 37916646.
